# Cutibacterium acnes (Propionibacterium acnes) 16S rRNA Genotyping of Microbial Samples from Possessions Contributes to Owner Identification

**DOI:** 10.1128/mSystems.00594-19

**Published:** 2019-11-26

**Authors:** Jiayue Yang, Tomoya Tsukimi, Mia Yoshikawa, Kenta Suzuki, Tomoki Takeda, Masaru Tomita, Shinji Fukuda

**Affiliations:** a Institute for Advanced Biosciences, Keio University, Tsuruoka, Yamagata, Japan; b Systems Biology Program, Graduate School of Media and Governance, Keio University, Fujisawa, Kanagawa, Japan; c RIKEN BioResource Research Center, Tsukuba, Ibaraki, Japan; d Faculty of Environment and Information Studies, Keio University, Fujisawa, Kanagawa, Japan; e Intestinal Microbiota Project, Kanagawa Institute of Industrial Science and Technology, Kawasaki, Kanagawa, Japan; f Transborder Medical Research Center, University of Tsukuba, Tsukuba, Ibaraki, Japan; g PRESTO, Japan Science and Technology Agency, Kawaguchi, Saitama, Japan; h Metabologenomics, Inc., Tsuruoka, Yamagata, Japan; University of California, San Diego

**Keywords:** *Cutibacterium acnes*, 16S rRNA genotype, skin microbiome, next-generation sequencing, owner identification

## Abstract

The human skin surface harbors huge numbers of microbes. The skin microbiota interacts with its host and forms a skin microbiome profile that is specific for each individual. It has been reported that the skin microbiota that is left on an individual’s possessions can act as a sort of “fingerprint” and be used for owner identification. However, this approach needs to be improved to take into account any long-term instability of skin microbiota and contamination from nonspecific bacteria. Here, we took advantage of single-nucleotide polymorphisms (SNPs) in the 16S-encoding rRNA gene of Cutibacterium acnes, the most common and abundant bacterium on human skin, to perform owner identification. We first developed a high-throughput genotyping method based on next-generation sequencing to characterize the SNPs of the C. acnes 16S rRNA gene and found that the genotype composition of C. acnes 16S rRNA is individual specific. Owner identification accuracy of around 90% based on random forest machine learning was achieved by using a combination of C. acnes 16S rRNA genotype and skin microbiome profile data. Furthermore, our study showed that the C. acnes 16S rRNA genotype remained more stable over time than the skin microbiome profile. This characteristic of C. acnes was further confirmed by the analysis of publicly available human skin metagenome data. Our approach, with its high precision, good reproducibility, and low costs, thus provides new possibilities in the field of microbiome-based owner identification and forensics in general.

**IMPORTANCE** Cutibacterium acnes is the most common and abundant bacterial species on human skin, and the gene that encodes its 16S rRNA has multiple single-nucleotide polymorphisms. In this study, we developed a method to efficiently determine the C. acnes 16S rRNA genotype composition from microbial samples taken from the hands of participants and from their possessions. Using the C. acnes 16S rRNA genotype composition, we could predict the owner of a possession with around 90% accuracy when the 16S rRNA gene-based microbiome profile was included. We also showed that the C. acnes 16S rRNA genotype composition was more stable over time than the skin microbiome profile and thus is more suitable for owner identification.

## Abstract (precis)

Cutibacterium acnes is the most common and abundant bacterial species on human skin, and the gene that encodes its 16S rRNA has multiple single-nucleotide polymorphisms. In this study, we developed a method to efficiently determine the C. acnes 16S rRNA genotype composition from microbial samples taken from the hands of participants and from their possessions. Using the C. acnes 16S rRNA genotype composition, we could predict the owner of a possession with around 90% accuracy when the 16S rRNA gene-based microbiome profile was included. We also showed that the C. acnes 16S rRNA genotype composition was more stable over time than the skin microbiome profile and thus is more suitable for owner identification.

## INTRODUCTION

Various microbes are found in and on our bodies, including the gut, the oral cavity, and our skin. The microbiota is site specific in and on our bodies and is unique to each individual (1). The human skin surface contains a complex microbial ecosystem, which is known as the skin microbiota (2). Skin microbiota interacts with its host through the host immune system and forms personally unique skin microbiome profiles in individuals (2–4). The skin microbiota persists on surfaces that come into contact with human skin (5). For example, the microbiota on an individual’s computer keyboard is similar to that of the skin microbiota of his/her fingers (5). Therefore, the skin microbiome profile could be used as a personal identification tool, similar to fingerprinting (5). In addition to the skin microbiome profile, other methods for skin sample-based personal identification have been proposed such as the use of skin metagenomic data (6, 7), skin microbiome minor populations (8), and even skin surface metabolites (9). However, the skin surface of the hand is easily contaminated during the activities of daily life (8), and the biomass of skin microbes is very small (10). In addition, within the skin microbiota, the major populations account for a large amount of the total biomass, whereas the less-abundant taxa, which may be more specific to each individual, represent a much smaller amount (6). These characteristics of skin microbes may make the skin microbiota easy to perturb (6). Furthermore, the metagenomic and metabolomic approaches mentioned above are costly and time-consuming and require large amounts of samples. There is thus plenty of room for improvement among the technologies that use skin samples for owner identification.

The use of single-nucleotide polymorphisms (SNPs) of one of the most common and abundant bacteria on human skin, the Gram-positive bacterium Cutibacterium acnes (former name, Propionibacterium acnes) (11–15), in such analyses may overcome these shortcomings. C. acnes begins to colonize the skin of prepubertal children at a very low level and increases in number throughout the teenage years until the individual is ∼20 years old (16). C. acnes, which prefers anaerobic conditions, accounts for 89% of the bacterial biomass in the human pilosebaceous units (12) and is widely known as the bacterium associated with acne (17). C. acnes species are divided into several distinct phenotypes (18–20). Studies that included skin metagenome analyses of various body parts suggested that the composition of C. acnes strains is individual specific and temporally stable (21, 22). A subsequent study using those skin metagenome data sets suggested that the presence or absence of features of the C. acnes pangenome may have the potential to predict its host (7). More importantly, the gene that encodes C. acnes 16S rRNA has many SNPs, which can be used to generate a strain-specific ribotype (12). C. acnes ribotype profiles from pilosebaceous units are diverse across individuals as revealed by full-length capillary sequencing analyses of the gene encoding 16S rRNA (12). Furthermore, there is a correlation between the C. acnes ribotype and the whole-genome genotype of C. acnes (23). Thus, we hypothesized that the ribotype of C. acnes may also be individual specific and may have the potential for personal identification.

Whereas capillary sequencing costs are much lower than those associated with metagenomic analysis, the former method is not suitable for analyzing large numbers of samples, as the process of cloning takes a substantial amount of time. As an alternative, we have developed a next-generation sequencing (NGS)-based high-throughput method for genotyping C. acnes 16S rRNA. This method enabled us to obtain the C. acnes ribotype profile of a large number of samples in a short period of time at low cost. In addition, we were able to detect the gene encoding C. acnes 16S rRNA by PCR from a small sample and to avoid the effects of the surrounding environment. We characterized the C. acnes ribotypes of healthy volunteers and examined these ribotypes over time with this method. We also used the C. acnes ribotype composition to carry out owner identification using the random forest machine learning. Finally, we confirmed our findings by using publicly available skin metagenomic data sets.

## RESULTS

### Development of an NGS-based C. acnes 16S rRNA genotyping method.

**FIG 1:**
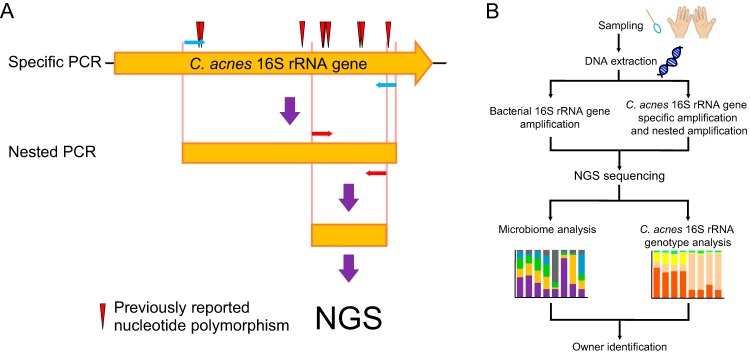
Experimental design. (A) PCR procedures to amplify the C. acnes 16S rRNA gene are shown. The blue and red arrows indicate the two sets of primers used for the gene-specific and variable-region-specific amplification reactions, respectively. (B) Schematic image of NGS-based skin microbiome and C. acnes 16S rRNA genotype analysis. Skin bacterial 16S rRNA gene amplification and amplification specifically for the C. acnes 16S rRNA gene were performed individually from the same DNA sample and analyzed by NGS.

We used an Illumina MiSeq sequencer to carry out the C. acnes 16S rRNA genotype analysis. As the MiSeq sequencer is unable to analyze the full-length C. acnes 16S rRNA gene, we chose a specific region of this gene for NGS analysis. In the C. acnes 16S rRNA gene, many nucleotide changes have been described that lead to the various ribotypes, and the area around V6 to V7 shows especially high nucleotide diversity (12). In this study, 6 of the 10 most abundant ribotypes contained one or two mutations within this region (12). Therefore, we designed primers that would specifically amplify the V6–V7 region of the C. acnes 16S rRNA gene (Fig. 1A). Then, the PCR products were used for library preparation, and NGS was performed. We used the extracted DNA not only for ribotyping of C. acnes strains but also for analysis of the 16S rRNA genes of the entire microbiome in the skin DNA samples (Fig. 1B).

### Analysis of the C. acnes 16S rRNA genotype from samples taken from hands and possessions.

**FIG 2:**
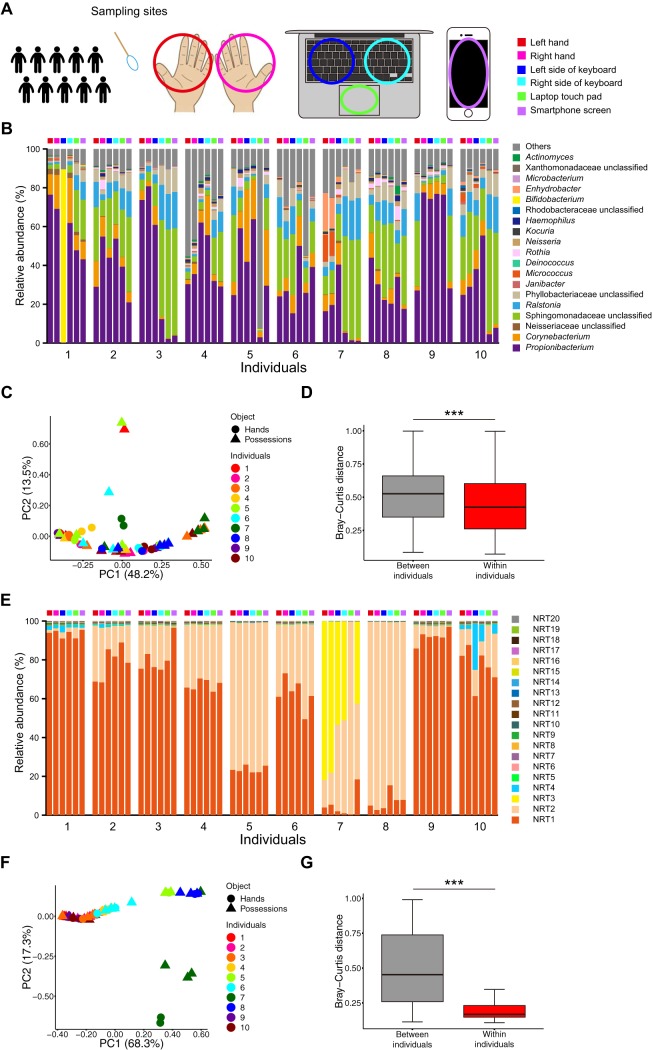
Skin microbiome and C. acnes NRT composition on hands and possessions. (A) Experimental design for the skin microbiome and C. acnes NRT composition analysis of 10 healthy volunteers. (B) Skin microbiome profile. The boxes above the graph represent sampling sites as shown in the key in panel A. (C and D) Bray-Curtis distances were used (C) to generate a PCoA of microbiome profiles and (D) to analyze the distances between and within individuals, which are shown as box plots. (E) Composition of the top 20 C. acnes NRTs among the hand and possession samples. Boxes above the graph indicate the sampling sites described in the panel B legend. (F and G) Bray-Curtis distances were used (F) to generate a PCoA of the whole C. acnes NRT compositions and (G) to analyze the distances between and within individuals, which are shown as box plots. ***, *P < *0.001 (Mann-Whitney *U* test).

We collected microbial samples from hands, keyboards, laptop touch pads, and smartphone screens from 10 volunteers (Fig. 2A) and performed both microbiome and C. acnes 16S rRNA genotype analyses. The microbiome analysis indicated that the microbiome profiles determined for skin samples and the corresponding possessions of each individual had a low degree of similarity (Fig. 2B). The similarity distribution of microbiome profiles between corresponding skin samples and possessions was further evaluated by using principal-coordinate analysis (PCoA) of Bray-Curtis distances (Fig. 2C). The distances among an individual’s possessions and hands were not much smaller than those between individuals and did not form clusters (Fig. 2C and D). This suggested that the similarity of the microbiome profiles of an individual’s hands and possessions is low. Qualitative and quantitative analysis using unweighted and weighted UniFrac distances showed similar results (see Fig. S1A to D in the supplemental material).

10.1128/mSystems.00594-19.1 FIG S1 Qualitative and quantitative analysis of skin microbiome and C. acnes NGS-detected ribotype (NRT) composition on hands and possessions. (A) PCoA of unweighted UniFrac distances for the skin microbiomes. (B) Comparisons of those distances across all samples between individuals and all samples within each individual were performed. (C) PCoA of weighted UniFrac distances of the skin microbiome. (D) Comparisons of these distances across all samples between individuals and all samples within each individual were performed. (E) PCoA of binary Euclidean distance of C. acnes NRT composition. (F) Comparisons of these distances across all samples between individuals and all samples within each individual were performed. (G) PCoA of Spearman correlation distances of C. acnes NRT composition. (H) Comparisons of these distances among all samples between individuals and all samples within individuals were performed. ***, *P < *0.001 (Mann-Whitney *U* test). Download FIG S1, EPS file, 1.5 MB. Copyright © 2019 Yang et al. 2019 Yang et al. This content is distributed under the terms of the Creative Commons Attribution 4.0 International license.

Next, we examined the 16S rRNA genotype composition for C. acnes with our method. As our sequencing approach could analyze only about a 400-bp region of the C. acnes 16S rRNA gene, the C. acnes ribotypes detected by this method were referred to as NGS-detected ribotypes (NRTs). BLAST analysis showed that, as expected, >93.7% of the operational taxonomic units (OTUs) detected were C. acnes (Fig. S2A and B). The OTUs that did not correspond to C. acnes were removed, and each unique 16S rRNA gene sequence of C. acnes was defined as an NRT. As this genotyping methodology is based on the many SNPs of the 16S rRNA gene, very high numbers of NRTs (*n* = 11,631) were detected by this method, indicating that this method is suitable for genotyping (Fig. S2C). We numbered each NRT according to its abundance among all the hand and possession samples, with the most abundant NRT referred to as NRT1. NRT1 through NRT3 accounted for >70% of the C. acnes NRT composition (Fig. S2D). Analyzing only the top 20 NRTs, their distribution patterns suggested similarities between the hand and possession samples for each individual (Fig. 2E). The similarity of the distributions among all C. acnes NRTs between skin samples and possessions was further evaluated by a PCoA using Bray-Curtis distances. Compared with the microbiome data, the samples for each individual formed clusters, and the distances between an individual’s possessions and hands were much smaller than those among individuals (Fig. 2F and G), indicating that the C. acnes NRT composition of an individual’s possessions resembles that of their owner. The qualitative and quantitative analysis performed using binary Euclidean distances and Spearman distances showed a similar result (Fig. S1E to H).

10.1128/mSystems.00594-19.2 FIG S2 C. acnes NRT analysis. (A and B) Composition of bacterial OTUs in (A) the bacterial 16S rRNA NGS dataset and (B) the C. acnes NRT NGS dataset. The “others” category refers to other 16S rRNA sequences. (C) Rarefaction curve of C. acnes NRT NGS data. (D) Composition of C. acnes NRTs among the C. acnes NRT NGS data. Download FIG S2, EPS file, 1.9 MB. Copyright © 2019 Yang et al. 2019 Yang et al. This content is distributed under the terms of the Creative Commons Attribution 4.0 International license.

**FIG 3:**
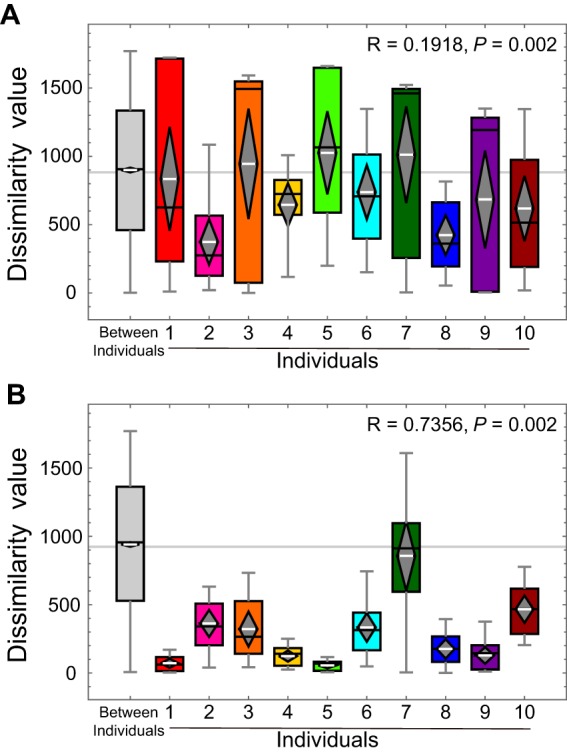
ANOSIM of skin microbiome and C. acnes NRT compositions between individuals and their possessions. (A and B) ANOSIM of (A) skin microbiome and (B) C. acnes NRT composition for each individual. Box plots were made using the Bray-Curtis distance matrix. The gray horizontal line shows the boundary of the lowest value of the 95% confidence interval from the all-samples data. In each box plot, the box represents the first and third quartiles, the diamond shows the entire range of the 95% confidence interval corresponding to the average value, the white line shows the average, and the black line shows the median value for each data set. The whiskers extend from the minimum value to the maximum value within each data set. In each graph, the box plot for the data representing distances between samples of different individuals was compared with that for each individual. Individuals for which the diamond (the entire range of the 95% confidence interval corresponding to the average value) is lower than the gray line (95% confidence interval corresponding to the data of distances between samples of different individuals) show similarity within that data set. The *R* value for the data shown in panel B was larger than that for the data shown in panel A, which indicates that the similarity of the C. acnes NRT compositions within each individual was higher than that of the microbiome. *P* values are also shown.

Because PCoA plots show only the two-dimensional information from each sample (Fig. 2C and F; see also Fig. S1A, C, E, and G), we further compared the component similarities of the 16S rRNA-based skin microbiome profile and the C. acnes NRT composition within each individual by analysis of similarity (ANOSIM) using Bray-Curtis distances. ANOSIM is an analysis method used to test whether there are significant differences among several groups of samples by using all dimensions of a PCoA (5). As a result, the correlation coefficient value (*R*) representing results of comparisons of C. acnes NRT composition between the hands and possessions of each individual was higher than for the skin microbiome profile, which indicated that the similarity of the C. acnes NRT compositions between the hands and possessions of each individual was higher than that of the skin microbiome profile (Fig. 3). These results demonstrated that the C. acnes 16S rRNA genotype composition of an individual has the potential of identifying owners of solid-surface, nonbiological samples.

### Owner identification using skin microbiota profile and C. acnes NRT composition.

**FIG 4:**
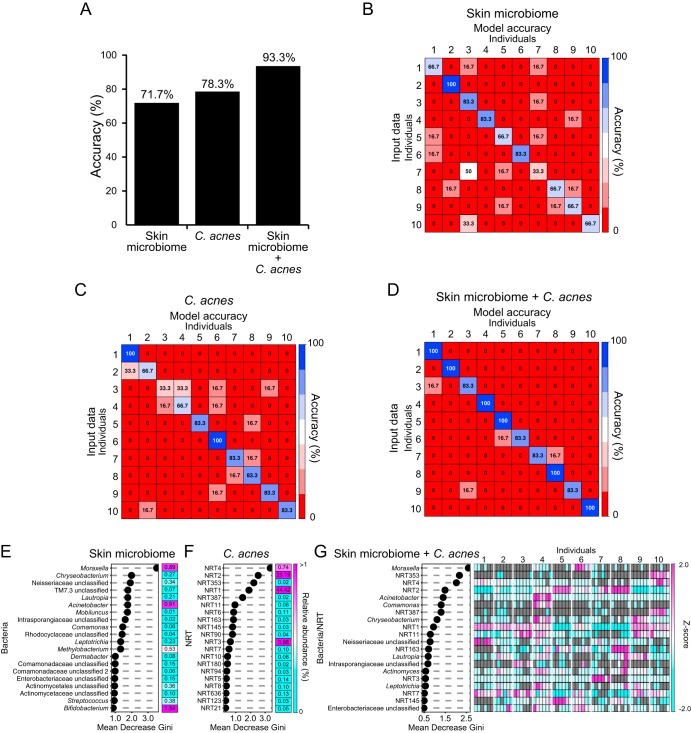
Owner identification based on the skin microbiome and C. acnes NRT composition using random forest machine learning. (A) Comparison of the accuracy results using the skin microbiome profile, the C. acnes NRT composition, and both sets of data. (B to D) Accuracy heat maps of owner identification using (B) the skin microbiome profile, (C) the C. acnes NRT composition, and (D) both sets of data are shown. Rows indicate the input data (fingers of both hands), and columns indicate the model accuracy results. (E to G) The Mean Decrease Gini of owner identification using (E) the microbiome profile and (F) the C. acnes NRT composition with a heat map of the average abundance of OTUs and using (G) both sets of data with a heat map showing the Z-scores is presented. In the Z-score heat map, gray rectangles indicate that the particular OTU was not detected in that sample. In panels E and F, the OTUs with an average abundance of >1% are shown in magenta, and the relative abundance value for each OTU is shown inside the boxes of the heat map.

On the basis of the results described above, we next attempted to use the C. acnes NRT composition of each individual for owner identification. We used the random forest algorithm with the skin microbiome profile and the C. acnes NRT composition to compare their accuracy rates. The accuracy rate determined using the skin microbiome profile was 71.7% (Fig. 4A and B). Using the C. acnes NRT composition, the accuracy increased slightly to 78.3% (Fig. 4A and C). Using a combination of C. acnes NRT composition and skin microbiome, the accuracy was increased to 93.3% (Fig. 4A and D). We performed cross-validation of the model accuracy, and the results were similar to those shown in Fig. 4A to D (see also Fig. S3). Therefore, it is unlikely that overfitting occurred. The top 20 components from the Mean Decrease Gini data from the random forest analysis, which represent the factors that contribute most to accuracy, are shown (Fig. 4E to G). The minor genera of an individual’s microbiome contributed to the accuracy of owner identification using the skin microbiome profile data (Fig. 4E), whereas both the major and minor members of the C. acnes NRT contributed to the accuracy of owner identification using the C. acnes NRT composition (Fig. 4F). Consistently, the minor genera of the skin microbiome and both the major and minor NRTs of C. acnes contributed to the accuracy of owner identification using the combination of microbiome data and C. acnes NRT composition (Fig. 4G). The Z-score heat map of the Mean Decrease Gini top 20 OTUs for this combination analysis showed that the abundances of those bacteria and of C. acnes NRTs occurred in a unique pattern in each individual, which likely contributed to the high accuracy (Fig. 4G).

10.1128/mSystems.00594-19.3 FIG S3 Cross-validation of owner identification based on the skin microbiome and C. acnes NRT composition using random forest machine learning. In cross-validation, five samples from an individual were used for model construction, and then the remaining sample was tested, followed by testing of all six different samples from that individual. Data representing averages of results from six trials performed using the six different sampling sites in each test are shown. (A) Comparison of the accuracy of the results using the skin microbiome profile, the C. acnes NRT composition, and both sets of data. (B to D) Accuracy heat maps of owner identification using (B) the skin microbiome profile, (C) the C. acnes NRT composition, and (D) both sets of data are shown. Rows indicate the input data (fingers of both hands), and columns indicate the prediction results. Download FIG S3, EPS file, 1.6 MB. Copyright © 2019 Yang et al. 2019 Yang et al. This content is distributed under the terms of the Creative Commons Attribution 4.0 International license.

### Temporal stability of the skin microbiome profile and C. acnes NRT composition.

**FIG 5:**
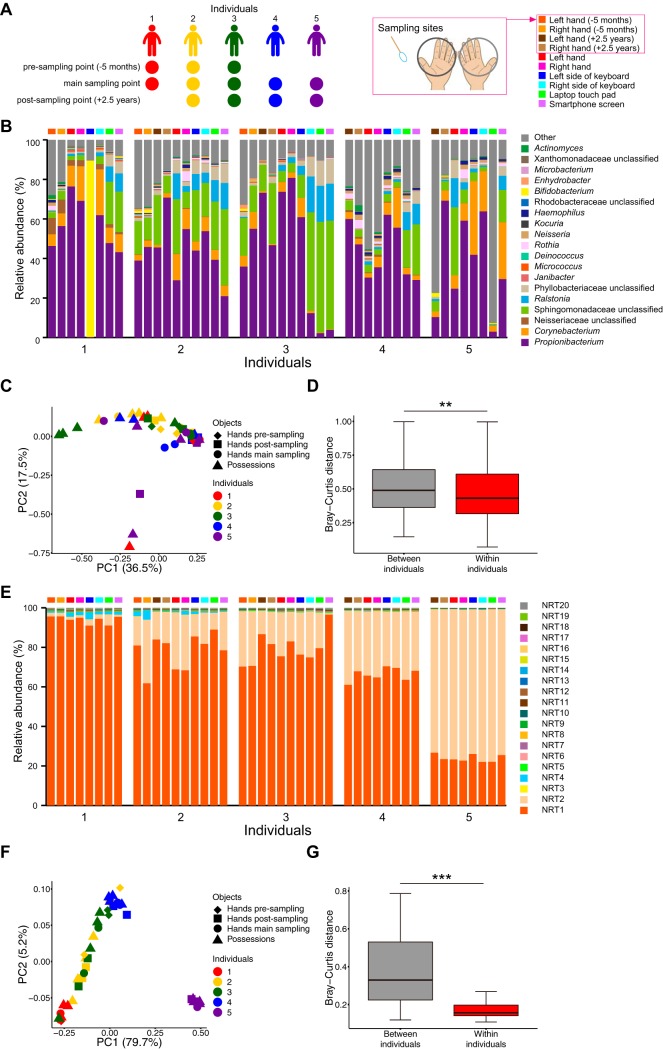
Assessment of the long-term stability of the skin microbiome and C. acnes NRT composition from hand samples. (A) Experimental design of the skin microbiome and C. acnes NRT composition analysis using long-term samples. (B) Skin microbiome profile of main sampling and pre- and postsampling of individuals 1 to 5. The boxes above the graphs represent the sampling sites as shown in panel A. (C and D) Bray-Curtis distances were used (C) to generate a PCoA of microbiome profiles and (D) to analyze the distances between and within individuals, which are shown as box plots. (E) Top 20 NRTs that make up the C. acnes NRT composition in the main sampling and pre- and postsampling of individuals 1 to 5. The boxes above the graphs represent the sampling sites as shown in panel A. (F and G) Bray-Curtis distances were used (F) to generate a PCoA of the whole C. acnes NRT compositions and (G) to analyze the distances between and within individuals, which are shown as box plots. **, *P < *0.01; ***, *P < *0.001 (Mann-Whitney *U* test).

We compared the stability of the skin microbiome and C. acnes NRT composition over time using sampled DNA from the hands of individuals. To assess the temporal stability of skin bacteria, we analyzed the microbiome profile and C. acnes NRT composition using DNA samples collected from the hands of individuals 5 months before (presampling point), the main sampling time point assessed above, and 2.5 years later (postsampling time point) (Fig. 5A). DNA samples were collected from three individuals (individuals 1 to 3) at the presampling time point and from four individuals (individuals 2 to 5) at the postsampling time point (Fig. 5A). The skin microbiome analysis suggested that the microbiome profiles at each sampling point varied for each individual (Fig. 5B). The similarity distribution of these skin microbiome profiles was further evaluated by a PCoA of Bray-Curtis distances (Fig. 5C and D). The plot from the PCoA shows that the samples collected at the pre- and postsampling time points for a given individual were separated from his/her main samples, which indicated that the components of the skin bacterial species were not stable over time (Fig. 5C). Distances between an individual’s long-term samples and main samples were not much smaller than those between individuals and did not form clusters (Fig. 5C and D), confirming that the similarity between an individual’s main samples and long-term samples was low. The unweighted and weighted UniFrac analyses showed similar results (Fig. S4A to D), which were consistent with a previous finding (6). Next, we evaluated the C. acnes NRT composition over a long-term interval. The composition of the top 20 NRTs suggested that the overall NRT data were similar among the main samples and the presampling and postsampling time points within the same individual (Fig. 5E). In a further evaluation with a PCoA of Bray-Curtis distances, the samples from each individual formed clusters, and the distances among an individual’s long-term samples and main samples were much smaller than those between individuals (Fig. 5F and G). The PCoA using binary Euclidean distance and Spearman distance analysis showed similar results (Fig. S4E to H). Therefore, the C. acnes NRT composition might be more stable than the skin microbiome profile within each individual.

10.1128/mSystems.00594-19.4 FIG S4 Qualitative and quantitative comparison of skin microbiome and C. acnes NRT composition of long-term samples from the hands with that of the main samples. (A) PCoA of unweighted UniFrac distances of the skin microbiome. (B) Comparisons of these distances among all samples between individuals and all samples within individuals were performed. (C) PCoA of weighted UniFrac distances of the skin microbiome. (D) Comparisons of these distances among all samples between individuals and all samples within individuals were performed. (E) PCoA of binary Euclidean distances of C. acnes NRT composition. (F) Comparisons of these distances among all samples between individuals and all samples within individuals were performed. (G) PCoA of Spearman correlation distances of C. acnes NRT composition. (H) Comparisons of these distances among all samples between individuals and all samples within individuals were performed. *, *P < *0.05; ***, *P < *0.001 (Mann-Whitney *U* test). Download FIG S4, EPS file, 1.5 MB. Copyright © 2019 Yang et al. 2019 Yang et al. This content is distributed under the terms of the Creative Commons Attribution 4.0 International license.

**FIG 6:**
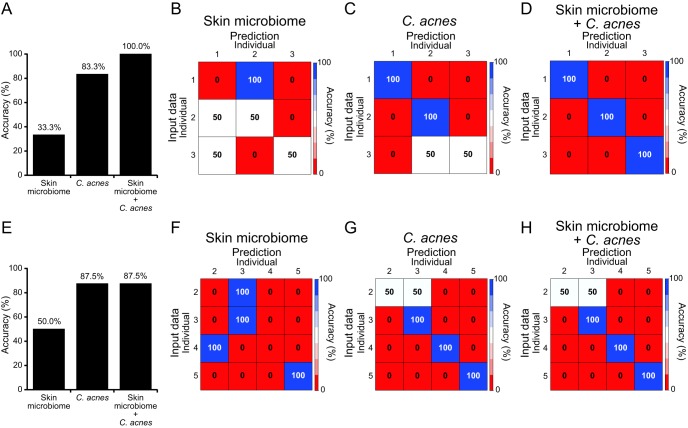
Prediction of owners using skin microbiome and C. acnes NRT composition data from different time points. (A) Comparison of the accuracy of the predictions using presampling data (from 5 months before the main sampling) from individuals 1 to 3. (B to D) Individual heat maps of owner prediction accuracy with presampling data using (B) the skin microbiome profile, (C) the C. acnes NRT composition, and (D) the two combined are shown. (E) Comparison of the accuracy of the prediction using postsampling data (2.5 years after the main sampling) from individuals 2 to 5. (F to H) Individual heat maps of owner prediction accuracy with postsampling data using (F) the microbiome profile, (G) the C. acnes NRT composition, and (H) the two combined are shown. Rows indicate the input data (from fingers of both hands), and columns indicate the prediction results.

On the basis of our findings that the C. acnes NRT composition profile is more suitable for owner identification over time than the skin microbiome profile, we thus carried out owner identification by using the long-term hand samples to predict the owner of sampled possessions. The accuracy rate of prediction using C. acnes NRT composition from presampling data was much higher than that of the microbiome profile (Fig. 6A to C and E to G). We also performed owner identification by using the combination of the C. acnes NRT composition and the skin microbiome profile from presampling data. Similarly to the findings with the main sampling data sets, the accuracy improved compared with the accuracy seen using C. acnes NRT composition only (Fig. 6A and D). Next, we carried out owner identification using postsampling data. As we had found with the presampling samples, the accuracy using C. acnes NRT composition was much higher than that of the skin microbiome profile (Fig. 6E to G). Using both the C. acnes NRT composition and the skin microbiome profile from postsampling data for owner identification, however, the accuracy was the same as that found by using C. acnes NRT composition only (Fig. 6E and H). These data suggested that the C. acnes NRT composition has greater potential for owner identification after a long period of time (up to 2.5 years) and that use of the combination of the C. acnes NRT composition and the skin microbiome profile may, under certain conditions, lead to increased accuracy.

### Owner identification using public data set of C. acnes 16S rRNA gene SNPs.

Finally, we examined the extent to which the long-term stability of C. acnes 16S rRNA gene SNPs is universal using publicly available skin metagenome data sets from Oh et al. (22). These data sets consist of skin samples from 17 body sites of 12 healthy volunteers that were collected at three time points over short (5-to-10-week) to long (10-to-30-month) sampling durations. In this analysis, we used the data sets corresponding to samples from the hypothenar region of the palm and extracted the C. acnes 16S rRNA gene reads to analyze the SNPs in each individual.

**FIG 7:**
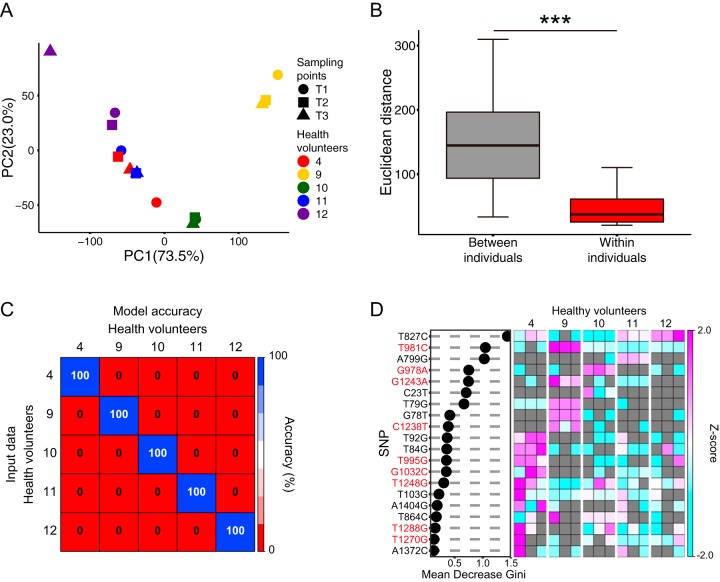
Owner identification using C. acnes 16S rRNA gene SNPs from publicly available metagenomic data sets. (A) PCoA of Euclidean distances and (B) comparisons of the distances between and within individuals for the C. acnes 16S rRNA gene SNPs. T1, T2, and T3 represent three different sampling points. From T1 to T2, the period was 10 to 30 months; from T2 to T3, the period was 5 to 10 weeks. (C) A heat map of owner prediction accuracy based on publicly available skin metagenomic samples using C. acnes 16S rRNA gene SNPs. (D) Mean Decrease Gini of owner identification using C. acnes 16S rRNA gene SNPs and a heat map of Z-scores are presented. The SNPs in red represent the SNPs within the area that was amplified by our nested PCR primer sets. In the Z-score heat map, gray rectangles indicate that the particular SNP was not detected in that sample. ***, *P < *0.001 (Mann-Whitney *U* test).

As this metagenomics data set contained not only reads from the region of the C. acnes 16S rRNA gene that we used here for owner identification but also reads from other regions, we decided to use the SNPs in the full-length 16S rRNA sequence of C. acnes to do the analysis. We used the full-length 16S rRNA gene sequence of C. acnes strain ATCC 6919 as the reference sequence. As several samples had a low number of reads after the mapping process, we included data only from individuals whose samples from all three time points had >1,500 reads in the following analysis. The PCoA of Euclidean distances and comparisons of the distances between and within individuals suggested that each individual had a unique and stable SNP pattern over time (Fig. 7A and B). Five of the 10 most abundant SNPs were found in the area amplified by the nested PCR primers that targeted the C. acnes 16S rRNA gene used in our analysis (Table S1 in the supplemental material), which suggested that this area may have a high mutation frequency. To further examine the long-term stability of these SNP patterns, we used the SNP patterns from all three time points from each individual to carry out owner identification by the random forest method. The model accuracy was 100% (Fig. 7C). The cross-validation of the model accuracy showed the same result (data not shown). Additionally, 9 of the top 20 SNPs that contributed to the Mean Decrease Gini were found in the area of C. acnes 16S rRNA amplified by our nested PCR primers (Fig. 7D), suggesting that this area is important for setting the criteria for each individual. The analysis described above further confirms that the C. acnes NRT composition is specific for each individual and has long-term stability.

10.1128/mSystems.00594-19.5 TABLE S1 Top 10 SNPs detected in skin metagenomic data. Download Table S1, DOCX file, 0.01 MB. Copyright © 2019 Yang et al. 2019 Yang et al. This content is distributed under the terms of the Creative Commons Attribution 4.0 International license.

## DISCUSSION

We have developed an NGS-based C. acnes 16S rRNA genotype-targeted approach to carry out owner identification. This method enabled us to determine the C. acnes NRT composition of individuals in a high-throughput manner and to obtain skin microbiome profile information simultaneously. Our analyses of C. acnes NRT composition from 10 individuals here and from publicly available skin metagenomic data confirmed the specificity at the individual level and the long-term stability of C. acnes 16S rRNA sequence SNPs. This specificity may be universally shared among different ethnic groups, as the healthy volunteers in our study were Japanese and the participants in the previous work were American (22). C. acnes is localized not only on the skin surface but also in the sebaceous glands, hair follicles, and pores (12). In addition, C. acnes bacteria form biofilms (24). Therefore, it is suggested that there might be tight cross talk between C. acnes and the host environment. Among all the human commensal microbes, it has been widely reported that gut microbiomes have long-term stability, which is due in part to the interaction with the host immune system (25–27). Similarly to the gut microbiota, the skin microbiota also maintains its homeostasis through interactions with the host immune system (4). Therefore, there is a possibility that these characteristics of C. acnes contribute to maintaining the similar C. acnes SNP compositions across samples from the same individual.

Recent studies have attempted personal identification using bacteria from human skin. In those studies, the skin microbiome was evaluated using 16S rRNA gene sequencing or metagenomic analysis (5, 6, 8). In a previous study using skin metagenome data, the accuracy rate was between 69% and 85% (6). In another report using minor components of the skin microbe population, the accuracy was between 78% and 95% (8). In a study that used the presence and absence of features of the C. acnes pangenome or the nucleotide diversities of clade-specific markers that were obtained from skin metagenome data from various body parts, the accuracy was between 30% and 100% (7). In our study, random forest machine learning-based owner identification was performed using both C. acnes and skin microbiome data. The accuracy rates using the microbiome and C. acnes NRT composition individually were 71.7% and 78.3%, respectively (Fig. 4A to C). In contrast, using both data sets increased the accuracy to 93.3% (Fig. 4A and D). The lower accuracy of the microbiome-based approach could have been due to the fact that the skin microbiota is more unstable over a long time period than C. acnes populations (Fig. 5) and that it has a low microbial biomass and is susceptible to contamination from nonspecific bacteria (8, 10). Although metagenome and metabolome analyses can be highly accurate (6, 7, 9), many pretreatment steps are needed and a computer server is necessary for the analysis of large numbers of samples. Furthermore, both of the methods require a large sample amount. The cost of sequencing 16S rRNA-encoding gene amplicons is much lower than the cost of metagenome and metabolome analyses, and the analysis procedure is much simpler and faster. It is, however, difficult to overcome the risk of contamination. With our C. acnes NRT analysis, we amplified specifically a region of the 16S rRNA gene of C. acnes, which helped us to obtain data from low-biomass samples and to overcome the risk of contamination without increased costs. In fact, the left side of the keyboard of individual 1 seems to have been contaminated with unspecific bacteria (i.e., *Bifidobacterium*), which resulted in a microbiome profile for this sample that was much different from that of its owner (Fig. 2B). Also, many samples seem to have had the previously reported kit contaminant OTUs in their microbiome profile (Fig. 2B; see also Fig. 5B) (28). Nevertheless, the C. acnes NRT composition from those samples remained highly similar to that from its owner (Fig. 2E; see also Fig. 5E), which suggests that this C. acnes NRT-targeted analysis has the potential to overcome bacterial contamination from daily life. In addition, this C. acnes NRT-targeted analysis may overcome the issue of long-term instability of skin microbiota, as the accuracy of owner prediction using the C. acnes NRT composition of long-term samples was much higher than that obtained using the skin microbiome profile (Fig. 6).

Interestingly, the accuracy of owner identification using the skin microbiome profile from each individual at the main time point was only slightly lower than that obtained using the C. acnes NRT composition, despite the PCoA and ANOSIM results showing that the differences between the microbiome data from the skin and possessions of the same individual were greater than the differences in the data determined for the C. acnes NRT composition. We believe that this was due to the contribution of the individual-specific minor bacterial population in each individual (Fig. 4E). As previously suggested, the minor population of skin bacteria may function as an individual-specific signature and may thus contribute to owner identification (8, 22). On the basis of the Z-score heat maps, the minor abundant bacteria/NRTs such as NRT353 and NRT387 in individual 10 or *Comamonas* in individual 4 and *Moraxella* in individual 6 may work as personal signatures, whereas the ratio of the major abundant bacteria/NRTs such as Acinetobacter and NRT1 to NRT3 in each individual may work together as the criteria to determine the individuals and may contribute to owner identification (Fig. 4G).

In conclusion, our results indicated that C. acnes 16S rRNA genotypes are unique within individuals and are relatively stable over time, both of which might contribute to owner identification of solid-surface, nonbiological samples. These findings suggest that, like fingerprints, the C. acnes 16S rRNA genotype has the potential to be a tool of forensic identification. As skin bacteria are easily obtainable from possessions, our approach is considered to be a useful tool for developing methods using bacterial samples from skin for owner identification.

## MATERIALS AND METHODS

### Sample collection.

In this study, samples were collected from 10 healthy Japanese volunteers 20 to 24 years of age. All volunteers were informed of the purpose of this study and signed a consent form. Samples were collected from four men and six women. There were two skin sites for sample collection (the fingers of the right hand and the fingers of the left hand) and four possession sites (the right side of the keyboard and the left side of the keyboard, the laptop touch pad, and the smartphone screen), all of which were sampled from each volunteer. The skin surface and possessions were sampled using sterilized swabs premoistened with TE10 buffer (10 mM Tris-HCl, 10 mM EDTA, pH 8) for 30 s, and the swab tips were transferred to sampling tubes filled with TE10 buffer and were shaken for 5 s; the swab tips were then discarded. Long-term samples were collected 5 months before and 2.5 years after the main sampling point. All samples were stored at −80°C until DNA extraction.

### DNA extraction.

Samples were first incubated with 15 mg/ml lysozyme (Wako Ltd.) at 37°C overnight. Next, the lysates were further incubated with achromopeptidase (Wako Ltd.) at a final concentration of 600 U/ml at 37°C for 8 h. Then SDS and proteinase K (Merck Millipore Ltd.) were added to reach final concentrations of 1% and 1 mg/ml, respectively, and the samples were incubated at 55°C overnight. After that, bacterial genomic DNA was purified from each sample according to the standard phenol-chloroform/isoamyl alcohol protocol as previously described (29).

### Microbiome 16S rRNA gene sequencing.

16S rRNA genes from the skin surface and possession samples were analyzed using a MiSeq sequencer (Illumina Inc.) and a previously described method (29). Briefly, to analyze the microbiome, PCR using DNA from each sample was performed with Tks Gflex DNA polymerase (TaKaRa Bio Inc.) and primers SP1-27Fmod and SP2-338R, which correspond to bacterial universal primer set 27Fmod/338R and contain the Read1/Read2 sequencing primer (Rd1/Rd2 SP) sequence (see Table S2 in the supplemental material) and amplify the V1 and V2 regions of 16S rRNA genes (30). The amplification process consisted of one denaturation step at 94°C for 1 min, followed by 28 cycles of 98°C for 10 s, 55°C for 15 s, and 68°C for 30 s, with a final extension step at 68°C for 3 min. The resulting reaction product was purified using Agencourt AMPure XP (Beckman Coulter Ltd.) and further amplified using the forward primer and reverse primer, which contain a unique 8-bp barcode sequence for each sample (indicated with an “N” in Table S2) and Rd1/Rd2 SP (16). After purification using Agencourt AMPure XP, a mixed sample was prepared by pooling equal amounts of PCR amplicons from all samples to make a final concentration of 4 nM. Libraries were then sequenced with 2 × 300-bp paired-end reads on the MiSeq sequencer.

10.1128/mSystems.00594-19.6 TABLE S2 Primers used in this study. Download Table S2, DOCX file, 0.01 MB. Copyright © 2019 Yang et al. 2019 Yang et al. This content is distributed under the terms of the Creative Commons Attribution 4.0 International license.

### C. acnes 16S rRNA genotyping sequencing.

Genotyping of C. acnes 16S rRNA genes from the skin surface and possession samples was carried out with the MiSeq sequencer. Using a method identical to that used for the microbiome analysis, PCR using DNA from each sample was performed with Tks Gflex DNA polymerase. First, the C. acnes 16S rRNA gene-specific PCR was run using primers CA_F (31) and CA_R, which target the region of C. acnes 16S rRNA corresponding to bp 414 to 1445 (Table S2). The PCR consisted of one denaturation step at 94°C for 5 min, followed by 30 cycles of 96°C for 10 s, 64°C for 15 s, and 68°C for 1 min, with a final extension step at 68°C for 2 min. The PCR products were diluted 300-fold in TE buffer, and then nested PCR was performed using primers CAV_F and CAV_R to amplify the variable region around V6 to V7 (bp 948 to 1334) from the specific PCR products. The nested PCR consisted of one denaturation step at 94°C for 2 min, followed by 20 cycles of 96°C for 10 s, 64°C for 15 s, and 68°C for 30 s, with a final extension step at 68°C for 2 min. Then, to incorporate the sample-specific 8-bp barcode sequences and perform NGS analysis of the microbiome samples simultaneously, Rd1/Rd2 SP sequences were added to the 5′ and 3′ ends of the nested PCR products by an additional round of PCR using SP1-950F and SP2-1334R primers. The PCR consisted of one denaturation step at 94°C for 2 min, followed by five cycles of 96°C for 10 s, 64°C for 15 s, and 68°C for 30 s, with a final extension step at 68°C for 2 min. Finally, the forward primer and reverse primer containing unique 8-bp barcode sequences were added by PCR using the same procedure as described above for the microbiome PCR. After that, all products were processed using the same method for the analysis of 16S rRNA gene sequences.

### Analysis of microbiome profile.

Sequencing for the microbiome profiles generated 3,241,784 paired-end reads obtained from 74 DNA samples. For the analysis of microbiomes, Fast Length Adjustment of Short Reads (FLASH) (v1.2.11) (32) was used to assemble the paired-end reads. For quality control, 30 bp of the 3′ end sequences were deleted before FLASH was used, and sequences that had a mean quality score of <25 and were >1,000 bp or <200 bp were discarded after FLASH by the use of an in-house script. Among the NGS samples, the maximum number of sequences was 48,512 reads per sample and the minimum number of sequences was 12,281 reads per sample. Therefore, for the microbiome analysis, 12,281 reads were selected randomly from each sample that had ≥12,281 reads, and these were processed using the Quantitative Insights Into Microbial Ecology (QIIME) (v1.9.1) pipeline (33). The reads were clustered into OTUs using 97% sequence similarity for the microbiome analysis, and they were then assigned using the UCLUST method (34).

### Analysis of C. acnes NRT composition.

In the C. acnes 16S rRNA genotyping sequencing, 2,969,625 paired-end reads were obtained from 74 DNA samples. FLASH and the same quality control procedures as described above were also used for assembling the C. acnes 16S rRNA genotyping sequencing data. For the C. acnes NRT analysis, reads that passed the quality check were selected randomly from each sample by the use of an in-house script and processed using the QIIME pipeline. Reads were then clustered into OTUs using 100% sequence similarity and were assigned to taxonomy with the Basic Local Alignment Search Tool (BLAST+) (v2.6.0) (35, 36). OTUs that did not correspond to C. acnes were removed, and singletons were removed to avoid sequencing errors. The number of reads that passed BLAST analysis per sample ranged from 31,900 to 10,000. Therefore, 10,000 reads were selected randomly from each sample again. These reads were finally clustered into OTUs, again using 100% sequence similarity. NRTs were then named according to their abundance across all samples, with the most abundant NRT referred to as NRT1.

### Owner identification.

For owner identification analyses, C. acnes NRTs and microbiome OTUs with an abundance of >0.01% were extracted from each sample. A random forest method was then implemented with the randomForest function in randomForest package v4.6-14 in R (37). In each test, 50,000 decision trees were generated. For the owner identification from the main time point samples, all samples from an individual were used. The classification model included data from the fingers of both hands and from the possessions, and the model accuracy was calculated from the out-of-bag (OBB) error rate. In cross-validation, five samples from each individual were used for model construction, which was then used to predict the owner of the remaining sample. The model construction and prediction were run upon all samples for each individual (six times in total per individual), and the average of the accuracy rates was calculated. For owner identification from the long-term samples, the classification model included data from the fingers of both hands and from the possessions of the owners from the main time point, and then the model was used to identify owners based on the presampling or postsampling data and the prediction accuracy was calculated. For the owner identification analysis performed using the publicly available skin metagenomic samples, the classification model included data from the samples collected from the hypothenar region of the palm for all three time points and the model accuracy was calculated from the OBB error rate. In the cross-validation of the publicly available data set, two samples from each individual were used for model construction, and then the remaining sample was used to predict the owner. The model construction and prediction were carried out with all samples from each individual (three times in total per individual), and the average of the accuracy rates was calculated.

### Processing of skin metagenomic data sets.

The data sets from the hypothenar region of the palm from the work performed previously by Oh et al. (22) (216 fastq files from 37 samples, 4,011,677,552 reads) were obtained from the National Center for Biotechnology Information Sequence Read Archive (NCBI SRA) using the SRA toolkit (https://www.ncbi.nlm.nih.gov/books/NBK158900/). The adaptor sequences were removed, and the reads with a Q score of <20 or with a length of <20 bp were excluded by the use of Trim Galore! (v0.4.4 dev) (https://www.bioinformatics.babraham.ac.uk/projects/trim_galore/). The remaining reads were mapped to the 16S rRNA gene sequence from the C. acnes strain ATCC 6919 using the Burrows-Wheeler alignment tool (BWA v0.7.17-r1188; option: BWA MEM with default parameters) (38), and the SNPs were analyzed by the use of SAMtools (v1.9) (38, 39). Each nucleotide that differed from C. acnes strain ATCC 6919 16S rRNA gene sequence was identified as a SNP. After that, taxonomy assignment was carried out with BLAST+ (v2.6.0) (E value, <1e−10) (35, 36); reads that contained insertions or deletions and reads that were soft or hard clipped as determined by SAMtools were removed. Finally, the individuals whose samples from all three time points contained >1,500 reads (five individuals, 15 samples) were chosen. From each sample, 1,845 reads were selected randomly and were included in the downstream analysis.

### Statistical analysis.

Distances among samples based on the Bray-Curtis, UniFrac, and binary Euclidean analyses were calculated by QIIME, and Spearman and Euclidean distances were calculated by R. The scatter plots and the box plots were drawn by R. ANOSIM was performed with the anosim function in vegan package v 2.5-1 in R (v3.4.2) (37).

### Use of human subjects.

This study was approved by the Ethics Committee of Keio University Shonan Fujisawa Campus (approval number 194).

### Data availability.

The C. acnes NRT and microbiome analysis data have been deposited in the DNA Data Bank of Japan (DDBJ) Sequence Read Archive (http://trace.ddbj.nig.ac.jp/dra/). The accession number for the C. acnes NRT analysis data is DRA008106. The accession number for the microbiome analysis data is DRA008105.
