# Supplementary material for: Cutibacterium acnes (Propionibacterium acnes) 16S rRNA Genotyping of Microbial Samples from Possessions Contributes to Owner Identification
Source: mSystems. 2019 Nov 26;4(6):e00594-19. doi: 10.1128/mSystems.00594-19 (PMC6880042; doi:10.1128/mSystems.00594-19)
Supplement: TABLE S1 [file mSystems.00594-19-st001.docx]

**TABLE S1** Top 10 SNPs detected in skin metagenomic data

| **SNP** | **Number of reads** |
| --- | --- |
| T827C | 1,085 |
| T981C | 659 |
| A1484G | 155 |
| G1243A | 133 |
| G1032C | 84 |
| T1248G | 82 |
| C23T | 81 |
| G978A | 47 |
| T103G | 37 |
| A1404C | 37 |

The SNPs shown in red are within the area amplified by the nested PCR primer set.
