# Supplementary material for: Cutibacterium acnes (Propionibacterium acnes) 16S rRNA Genotyping of Microbial Samples from Possessions Contributes to Owner Identification
Source: mSystems. 2019 Nov 26;4(6):e00594-19. doi: 10.1128/mSystems.00594-19 (PMC6880042; doi:10.1128/mSystems.00594-19)
Supplement: TABLE S2 [file mSystems.00594-19-st002.docx]

**TABLE S2** Primers used in this study

| **Name** | **DNA sequence (5'−3')** | **Source or reference** |
| --- | --- | --- |
| SP1-27Fmod | ACACTCTTTCCCTACACGACGCTCTTCCGATCTAGRGTTTGATYMTGGCTCAG | (1) |
| SP2-338R | GTGACTGGAGTTCAGACGTGTGCTCTTCCGATCTTGCTGCCTCCCGTAGGAGT | (1) |
| forward primer | AATGATACGGCGACCACCGAGATCTACACNNNNNNNNTATGGTAATTGTAGRGTTTGATYMTGGCTCAG | (1) |
| reverse primer | CAAGCAGAAGACGGCATACGAGATNNNNNNNNAGTCAGTCAGCCTGCTGCCTCCCGAGGAGT | (1) |
| CA_F | GGGTTGTAAACCGCTTTCGCCT | (2) |
| CA_R | TTCGACGGCTCCCCCACAAC | This study |
| CAV_F | AGAACCTTACCTGGGTTTGA | This study |
| CAV_R | GATCTGCGATTACTAGCGAC | This study |
| SP1-950F | ACACTCTTTCCCTACACGACGCTCTTCCGATCTAGAACCTTACCTGGGTTTGA | This study |
| SP2-1334R | GTGACTGGAGTTCAGACGTGTGCTCTTCCGATCTGATCTGCGATTACTAGCGAC | This study |

Nucleotides indicated by ‘N’ correspond to the sample-specific 8-bp barcode that was incorporated into the second round of microbiome PCR and *C. acnes* genotyping.

**References**

1. Murakami S, Goto Y, Ito K, Hayasaka S, Kurihara S, Soga T, Tomita M, Fukuda S. 2015. The Consumption of Bicarbonate-Rich Mineral Water Improves Glycemic Control. Evid Based Complement Alternat Med 2015:824395.

2. Sfanos KS, Isaacs WB. 2008. An evaluation of PCR primer sets used for detection of *Propionibacterium acnes* in prostate tissue samples. Prostate 68:1492-5.
